# High-temperature chlorination of PbO and CdO induced by interaction with NaCl and Si/Al matrix

**DOI:** 10.1039/c8ra06255b

**Published:** 2018-10-08

**Authors:** Xinye Wang, Hao Xie, Rong Du, Yuying Liu, Pingfang Lin, Jubing Zhang, Changsheng Bu, Yaji Huang, Wen Zhang

**Affiliations:** Jiangsu Provincial Key Laboratory of Materials Cycling and Pollution Control, School of Energy and Mechanical Engineering, Nanjing Normal University Nanjing 210042 Jiangsu China xinye.wang@njnu.edu.cn; Key Laboratory of Energy Thermal Conversion and Control of Ministry of Education, School of Energy and Environment, Southeast University Nanjing 210096 Jiangsu China heyyj@seu.edu.cn; Nanjing Shangyuan Industrial Gas Plant Nanjing 211100 Jiangsu China zw25102@163.com

## Abstract

Municipal solid-waste incineration leads to emission of lead (Pb) and cadmium (Cd), which vaporize in furnace and condense in flue. NaCl in waste has been proven to enhance volatilization of Pb and Cd at high temperatures *via* chlorination of oxides to chlorides; however, this process was not well-understood so far due to its complexity. This study decoupled the indirect chlorination process and direct chlorination process so that these two processes were investigated separately. A horizontal tube furnace was used to heat the mixtures of NaCl and Si/Al matrix for indirect chlorination and the mixtures of NaCl, PbO/CdO and Si/Al matrix for direct chlorination. A set of dynamic sampling devices was designed and used to obtain dynamic data during temperature rising. The indirect chlorination process was initiated above 800 °C in O_2_ + H_2_O atmosphere and O_2_ atmosphere and above 1000 °C in N_2_ atmosphere. Al_2_O_3_ exhibited higher activity than SiO_2_ to react with NaCl, releasing HCl or Cl_2_. In the Cl release reaction, NaCl was in the gas phase. The direct chlorination process was initiated at 650–700 °C when the Si/Al matrix contained SiO_2_ only and at around 800 °C when the Si/Al matrix contained Al_2_O_3_ only or both SiO_2_ and Al_2_O_3_. SiO_2_ exhibited higher activity than Al_2_O_3_ in direct chlorination. The pre-reaction between PbO/CdO and Si/Al matrices was considered as the necessary condition for direct chlorination. During chlorination in O_2_ + H_2_O atmosphere, indirect chlorination and direct chlorination occurred simultaneously, and the latter dominated the volatilization of Pb and Cd.

## Abstract 2

The chlorination process by NaCl was decoupled as indirect chlorination and direct chlorination, which were investigated separately.

## Graphical abstract

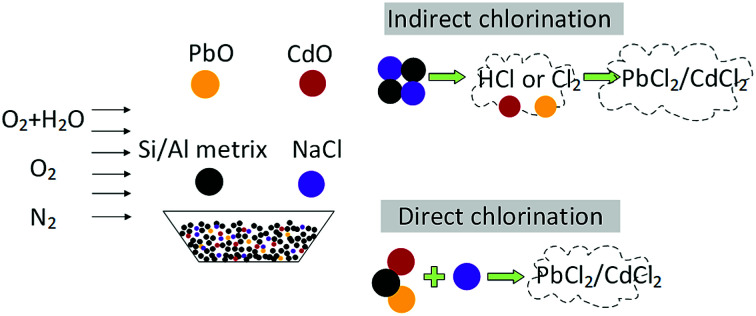

## 1. Introduction

Incineration has become a preferred method to dispose municipal solid waste (MSW) in the cities due to developed economy and dense population. There are at least 1179 MSW incineration plants around the world, and the total capacity exceeds 700 000 MT d^−1^.^1^ In developed European countries, about 50% of MSW is disposed by incineration, and the incineration capacity has almost reached saturation.^1^ In developing countries, the use of MSW incineration is increasing rapidly. As the largest developing country, China has both the largest scale and the fastest growth of incineration capacity.^2^ The “13th Five-Year Plan” (2016–2020) announced by the State Council of China requires that 40% of MSW should be incinerated before 2020.^3^ However, most developing countries have no sensible social mechanism of MSW classification, resulting in more heavy metals such as lead (Pb) and cadmium (Cd) in incinerators.^4^

In the combustion system, Pb and Cd are considered as semi-volatile metals, which evaporate from MSW in the furnace to flue gas and then condense as a solid phase.^5–7^ During this evaporation–condensation process, a small amount of Pb and Cd is converted to submicron aerosols (PM_1_), which cannot be captured by dedusting equipments with high efficiency.^6,7^ The escaping aerosols are very harmful to the human body.^8^

Chlorine (Cl) has been proven to be able to enhance the volatilization of Pb, Cd and other metals during combustion. The typical forms of Cl in MSW are polyvinyl chloride (PVC, organic Cl) and NaCl (inorganic Cl).^9^ In the previous research of fixed bed incineration, the addition of 1% Cl in the form of PVC or NaCl into simulated MSW caused an increase in the volatilization fractions of Pb and Cd from around 20% and 50% to around 80% and 90%, respectively.^10^ Most incineration researches using fixed bed, fluidized bed or kiln indicated a similar effect.^11–14^

The positive roles of PVC and NaCl in metal volatilization during incineration are usually interpreted by thermodynamic equilibrium investigation. Pb and Cd are considered to transform from oxides with a melting point of around 900 °C to chlorides with a melting point of around 500 °C in the presence of Cl during incineration according to the calculations based on Gibbs free energy minimization.^10,15–17^ Overall, chlorination is the key to the positive effects of PVC and NaCl on Pb and Cd volatilization.

There have been some investigations on the chlorination mechanisms of PVC. Wang *et al.* considered that the chlorination reaction between PVC and PbO was indirect.^18^ PVC released HCl at 225 °C; then, HCl reacted with PbO, producing PbCl_2_ and finally, PbCl_2_ volatilized above 500 °C.^18^ This process was described as indirect chlorination, which has been reported by other researchers.^10,19,20^ However, not all the PVC-metal oxide systems follow indirect chlorination. Kosuda *et al.* found direct chlorination between PVC and ZnO for the catalysis of ZnO.^21^

There is less research on the chlorination process between NaCl and metal oxides during incineration. The research group of Zhang and He conducted great studies on this topic.^22,23^ They used thermogravimetric analysis combined with differential scanning calorimetry (TG-DSC) to detect the chlorination of PbO and other metals by NaCl. It was found that NaCl alone reacted with PbO *via* a liquid–solid reaction but not with CdO, ZnO and CuO.^22^ The chlorination of PbO by NaCl was promoted in the presence of Al_2_O_3_ or SiO_2_.^23^ Many researchers have also used NaCl to remove heavy metals from ash successfully; however, few chlorination mechanisms were reported except some kinetic and dynamic studies.^24–26^

In the present study, further mechanisms of chlorination of PbO and CdO by NaCl were revealed to help the prediction and control of heavy metal emission during incineration. Unlike the experimental method (TG-DSC) used by the previous research, a dynamic sampling device was designed and used so that the dynamic release of chlorine and metals from the horizontal tube furnace was detected directly. Moreover, the tube furnace was more flexible regarding atmosphere selection (most TG cannot afford water vapour) and sample composition selection (samples are used in micrograms in TG so that the complex component cannot be prepared in stable proportions in different experiments). Therefore, the effects of water vapour and SiO_2_/Al_2_O_3_ molar ratio could be taken into consideration as well. The chlorination process was decoupled into indirect chlorination and direct chlorination, which were investigated separately. In indirect chlorination, first, NaCl releases Cl-containing gases (HCl or Cl_2_) and then, the Cl-containing gases chlorinate metal oxides.^27^ In direct chlorination, NaCl donates Cl to metals without releasing any gas.^27^

## 2. Materials and methods

### 2.1 Materials

During waste incineration, indirect and direct chlorination processes usually couple with each other. To decouple these two processes, the reaction materials were selected for different experimental cases.

The contents of reactants for indirect chlorination and direct chlorination are listed in Table 1, according to a previous research.^10^ SiO_2_, Al_2_O_3_, PbO, CdO and NaCl used were guaranteed reagents. Two steps were used for decoupling. In the first step, no PbO and CdO were added in the indirect chlorination experiments and chlorine release was used to represent the occurrence of indirect chlorination. The reactions between Cl-containing gas and heavy metal oxides were fast and easy; thus, when Cl-containing gas was produced, indirect chlorination occurred.^28^ Three atmospheres (N_2_, O_2_, and 85% O_2_ + 15% H_2_O) were compared in the indirect chlorination experiments. In the second step, only N_2_ atmosphere was used to avoid the release of Cl in direct chlorination experiments. Heavy metal release was used to evaluate the direct chlorination processes. The reducibility of combustible matter can enhance the volatilization of Pb and Cd.^10^ Consequently, carbon particles were excluded from the reaction system to ensure that chlorination was the only factor used to enhance metal volatilization besides temperature in direct chlorination experiments. Equally, carbon particles were excluded in indirect chlorination experiments. More details about decoupling are presented in Sections 4.1 and 4.4.

**Table 1:** Mass and mass fraction of reactants in the experimental cases

Case	Combustible matter	Incombustible matter	Chlorine	Heavy metal	
	Carbon particle (not added actually)	SiO_2_ + Al_2_O_3_^a^ (MP = 1650/2000 °C, BP = 2230/2977 °C, *d* < 0.5 mm)	NaCl^a^ (MP = 801 °C, BP = 1465 °C, *d* < 0.5 mm)	PbO^a^ (MP = 888 °C, BP = 1470 °C, *d* < 0.5 mm)	CdO^a^ (MP = 900 °C, BP = 1385 °C, *d* < 0.5 mm)
Indirect chlorination (N_2_, O_2_, O_2_ + H_2_O)	10.5 g (70%)	4.5 g (30%)	0.25 g (1% Cl)	0	0
Direct chlorination (N_2_)				2.42 mg (1500 mg Pb per kg)	25.7 mg (1500 mg Cd per kg)

a MP, melting point; BP, boiling point; *d*, particle size.

### 2.2 Experimental methods

Simulated incineration was carried out in a horizontal tube furnace with 500 mm inside diameter and 300 mm length of the temperature preservation zone. The gas flow rate was 500 mL min^−1^, and the heating rate was 10 °C min^−1^.

**Fig. 1:**
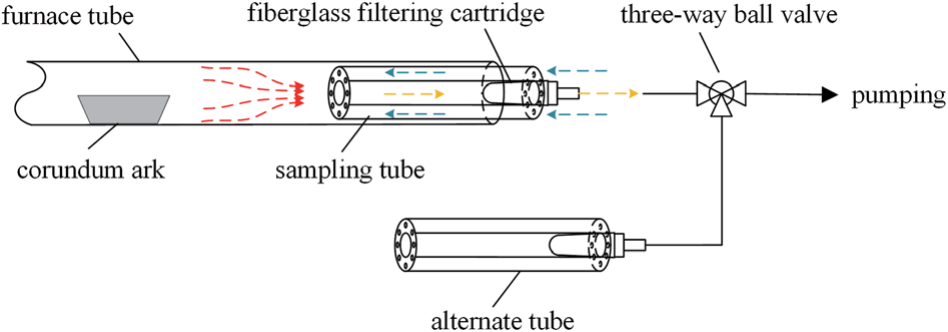
Heavy metal sampling device.

Traditional heavy metal sampling methods are filtering or adsorption outside a furnace at low temperature.^10,16^ With decreasing temperature, some amount of evaporated Pb and Cd can condense on the surfaces of the furnace exit and sampling pipeline, resulting in sampling loss. Therefore, the recovery rate is usually below 80% accompanied with several seconds of delay during sampling.^10,16^ A set of high-temperature sampling tubes (Fig. 1) were designed to capture evaporated Pb and Cd with high efficiency and good dynamic response. During metal sampling, the pumping flow rate was greater than the supplying flow rate; thus, the air outside was pumped in a reverse manner into the sampling tube, which cooled the sampling gas. The evaporated Pb and Cd partly condensed on the inner surface of the sampling tube. The remaining species were collected by a fiberglass filtering cartridge inserted at the end of the sampling tube. The sampling tube was replaced by a new one every 5 min.

During Cl sampling, the sampling tube was used as well to remove the evaporated NaCl, and the alternative absorption bottle with 1 mol L^−1^ NaOH solution was used to collect HCl or Cl_2_ gas. The atmosphere of 85% O_2_ + 15% H_2_O was generated by two sages of saturated vapour using O_2_ as the carrier gas, which was similar to the method used by Fraissler *et al.*^29^ The sampling time of each bottle was 10 min for dynamic Cl emission data.

### 2.3 Evaluation of Pb, Cd and Cl sampling method

The recovery rates of Pb and Cd were higher than 90% in each case, which was confirmed by detecting the metals in residues and sampling tubes. The recovery of Cl in each case was also higher than 90%, which was confirmed by detecting Cl in residues, sampling tubes and adsorption bottles.

### 2.4 Methods of Pb, Cd and Cl detection

The inner wall and the fiberglass filtering cartridge were washed with 5% HNO_3_ solution in direct chlorination. After diluting to a constant volume with water (18 MΩ cm), flame atomic absorption spectrophotometry was used to detect Pb and Cd concentrations (RSD < 2%). The solutions in adsorption bottles were diluted to a constant volume and detected by ion chromatography (RSD < 3%).

### 2.5 Methods of reaction product detection

The fractions of Na, Pb and Cd in products were too low to detect the components of these metals by X-ray diffraction (XRD) or some other methods. Therefore, more Na, Pb or Cd were added into the reaction system for product detection. Several molar ratios of reactants were used to prepare reaction samples, *i.e.*, Na/Si/Al = 1 : 2 : 0, 1 : 0 : 2 and 1 : 1 : 1 for indirect chlorination product preparation, whereas Pb/Cd/Si/Al = 1 : 1 : 2 : 0, 1 : 1 : 0 : 2 and 1 : 1 : 1 : 1 were used for direct chlorination products. It should be noted that the reagents used were all crystalline except Al_2_O_3_, which could not be detected by XRD.

## 3. Results

### 3.1 Cl release characteristics of NaCl

The Cl release was characterized by the change in the release fraction and release rate with increasing temperature. Three atmospheres (N_2_, O_2_, 85% O_2_ + 15% H_2_O) and three Si/Al molar ratios (1 : 0, 0 : 1 and 2 : 1) were used to reveal the effect of atmosphere and Si/Al matrix on Cl release.

**Fig. 2:**
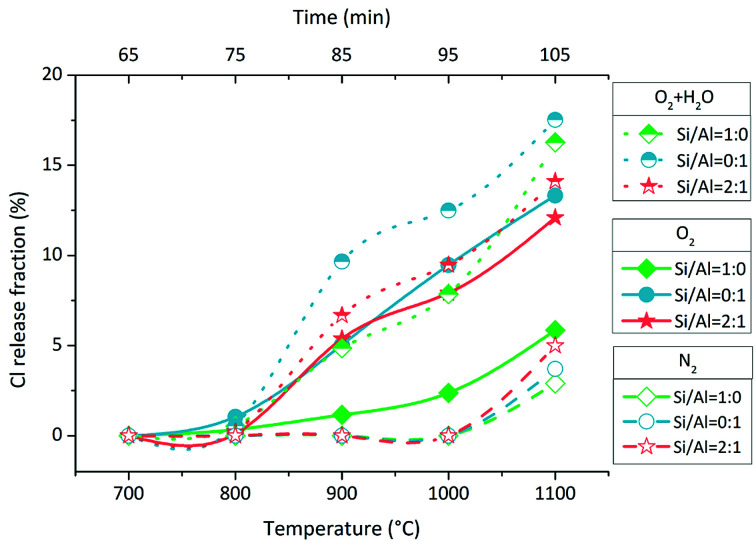
Accumulative release fractions of Cl during temperature rising.

In O_2_ atmosphere, Cl release was initiated at 700–800 °C with a small amount (Fig. 2, solid line and solid symbol). The Cl release fraction in the case of Si/Al = 0 : 1 was close to that in the case of Si/Al = 2 : 1 at each temperature, but it was clearly higher than that in the case of Si/Al = 1 : 0. When H_2_O was added to O_2_, producing 85% O_2_ + 15% H_2_O, Cl release was enhanced significantly in all three cases of different Si/Al molar ratios (Fig. 2, dot line and half up symbol). The initial temperature of Cl release was still 700–800 °C, and it was not lowered in the presence of H_2_O. In N_2_ atmosphere, Cl was released significantly above 1000 °C, and no Cl release occurred at 1000 °C (Fig. 2, dash line and hollow symbol). Overall, the positive effect of atmospheres on Cl release followed the sequence of O_2_ + H_2_O > O_2_ > N_2_. In all cases, the Cl release fractions were less than 20% when the temperature increased to 1100 °C.

**Fig. 3:**
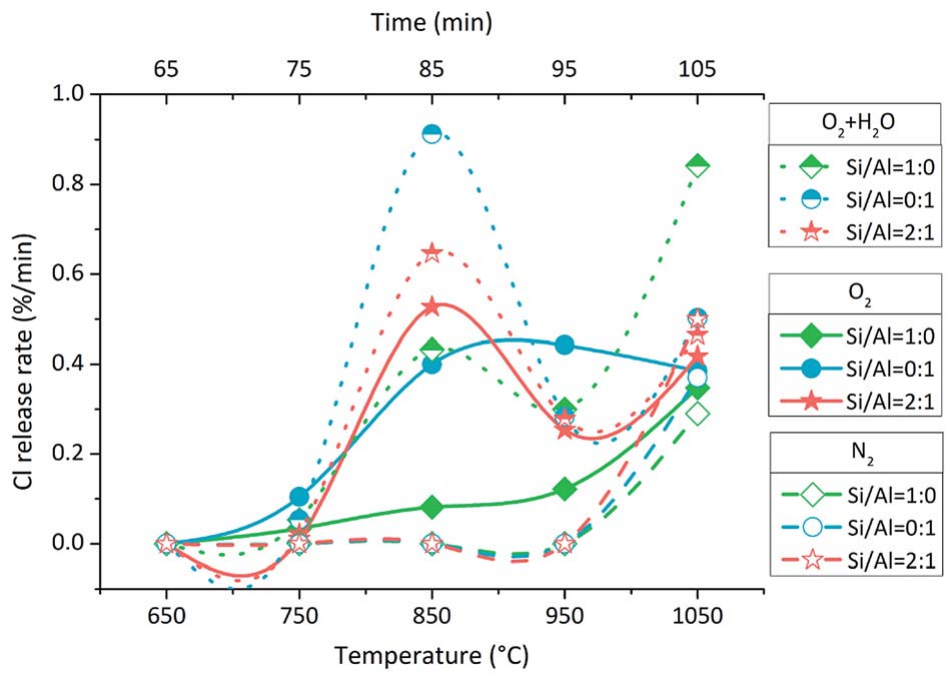
Cl release rates during temperature rising.

The Cl release rate was calculated by dividing the Cl amount in each sampling bottle by 10 min. There were two types of Cl release rate changes with increasing temperature (Fig. 3). The first type was the bimodal form or the tendency at least in O_2_ and O_2_ + H_2_O atmospheres except for the change with Si/Al = 1 : 0 in O_2_ atmosphere; this was the second type, which was similar to that in N_2_ atmosphere. In the bimodal form of Cl release rate change, one peak was at around 850 °C, whereas one valley was at around 950 °C. The Cl release rate followed different sequences in three atmospheres. In O_2_ + H_2_O atmosphere, the sequence was Si/Al = 0 : 1 > Si/Al = 2 : 1 > Si/Al = 1 : 0 at 850 °C and Si/Al = 1 : 0 > Si/Al = 2 : 1 ≈ Si/Al = 0 : 1 at 1050 °C. In O_2_ atmosphere, the sequence was Si/Al = 0 : 1 > Si/Al = 2 : 1 > Si/Al = 1 : 0 at 850 °C, and it was very close at 1050 °C. In N_2_ atmosphere, the change in the Cl release rate was simple, and the sequence was Si/Al = 2 : 1 > Si/Al = 0 : 1 > Si/Al = 1 : 0 at 1050 °C. In all cases, the Cl release rates were less than 1% min^−1^ when the temperature increased to 1100 °C.

### 3.2 Pb and Cd volatilization characteristics with NaCl addition in N_2_ atmosphere

**Fig. 4:**
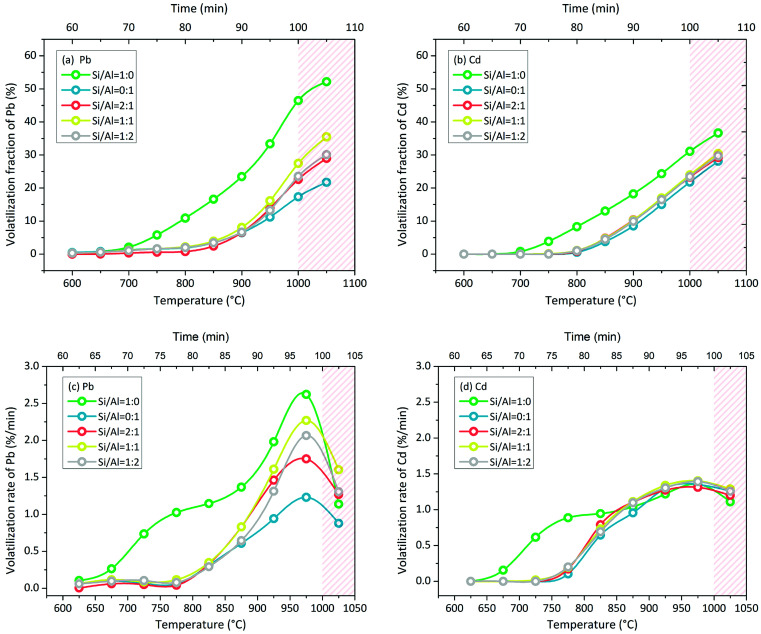
Accumulative volatilization fractions of (a) Pb and (b) Cd and volatilization rates of (c) Pb and (d) Cd with increasing temperature in N_2_ atmosphere. Red slash area indicates that the data inside were obtained in multi-factor coupling processes, whereas the rest of the area indicates that the data inside were obtained in the direct chlorination process only.

Pb volatilization with NaCl addition in N_2_ atmosphere was characterized by the change in the volatilization fraction and volatilization rate with increasing temperature. The green line of Si/Al = 1 : 0 is clearly different from other lines in Fig. 4(a). When the matrix consisted of SiO_2_ only without Al_2_O_3_ (Si/Al = 1 : 0), Pb volatilization was initiated at 650–700 °C. When the matrix consisted of Al_2_O_3_ in the presence or absence of SiO_2_, Pb volatilization was initiated at 800–850 °C. Moreover, the accumulative volatilization fraction of Pb in the case of Si/Al = 1 : 0 was always higher than that in other cases at each temperature; it was 52% at 1050 °C and the other values were in the range of 21–35%. Except for the case of Si/Al = 1 : 0, the other samples contained Al_2_O_3_ in the matrix, and the accumulative volatilization fractions were close to each other below 900 °C. Then, the volatilization fractions followed the sequence of Si/Al = 1 : 1 > Si/Al = 1 : 2 ≈ Si/Al = 2 : 1 > Si/Al = 0 : 1 above 900 °C.

The volatilization rate in the case of Si/Al = 1 : 0 was higher than that in other cases below 1000 °C (Fig. 4(c)); it was initiated at 650–700 °C and increased nonlinearly. With increasing temperature, the increase in the volatilization rate first slowed down at 750–850 °C and then speeded up at 850–1000 °C. When the temperature increased to 1050 °C, the volatilization rates decreased in all cases.

The volatilization characteristics of Cd with NaCl addition in N_2_ atmosphere were similar to that of Pb, but the volatilization process was weaker. The accumulative volatilization fraction of Cd in the case of Si/Al = 1 : 0 was higher than that in other cases as well (Fig. 4(b)). When the matrix consisted of Al_2_O_3_, the curves of the accumulative volatilization fraction and volatilization rate were close to each other at each temperature (Fig. 4(b) and (d)). Cd volatilization was initiated at around 700 °C in the case of Si/Al = 1 : 0 and at around 800 °C in other cases, which was similar to that observed for Pb volatilization.

The volatilization rate in the case of Si/Al = 1 : 0 was higher than that in other cases below 850 °C and then became the same as that in other cases above 850 °C (Fig. 4(c)); it was initiated at 650–700 °C and increased nonlinearly. With increasing temperature, the increase in the volatilization rate first slowed down at 750–850 °C and then speeded up at 850–1000 °C, which was the same as the observations for Pb volatilization. When the temperature was above 1050 °C, the volatilization rates decreased slightly in all cases.

### 3.3 Pb and Cd volatilization characteristics with NaCl addition in O_2_ + H_2_O atmosphere

**Fig. 5:**
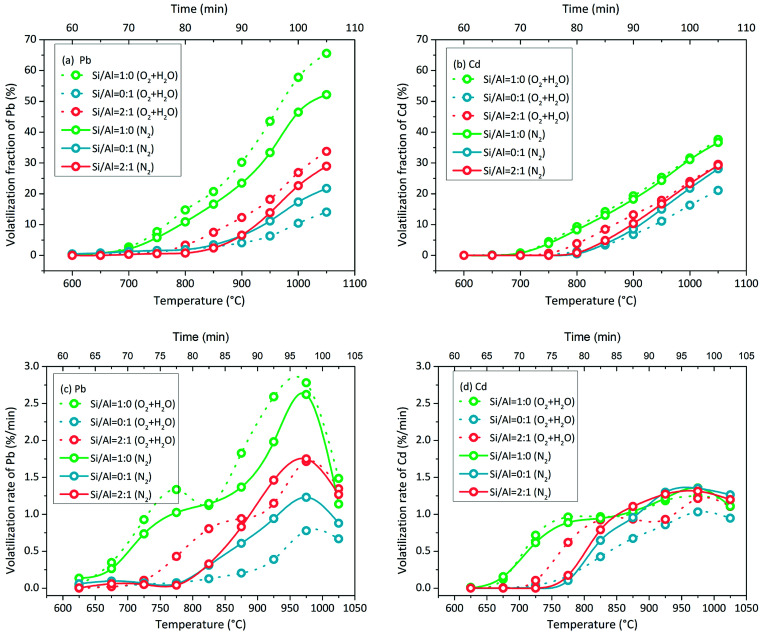
Accumulative volatilization fractions of (a) Pb and (b) Cd and volatilization rates of (c) Pb and (d) Cd with increasing temperature in 85% O_2_ + 15% H_2_O atmosphere and N_2_ atmosphere.

The comparison of the volatilization in O_2_ + H_2_O atmosphere and N_2_ atmosphere is shown in Fig. 5. When the Si/Al matrix consisted of SiO_2_, O_2_ + H_2_O was more favourable for Pb volatilization than N_2_. This effect was small for Cd volatilization. However, when the Si/Al matrix consisted of Al_2_O_3_ only, the O_2_ + H_2_O atmosphere inhibited both Pb and Cd volatilization. The initial temperature remained the same in SiO_2_-only case, whereas it was lowered from 800–850 °C to 750–800 °C in the SiO_2_ + Al_2_O_3_ case when the atmosphere was changed from N_2_ to O_2_ + H_2_O (Fig. 5(c) and (d)).

## 4. Discussion

### 4.1 Using Cl release to characterize indirect chlorination

Indirect chlorination consists of Cl release and metal chlorination. We used the first step of Cl release to characterize indirect chlorination for two reasons. The first reason is the second step of metal chlorination that occurs in direct chlorination as well. Therefore, metal volatilization was not the unique mark for indirect chlorination. The second reason is the reactivity between HCl/Cl_2_ and metal oxide, which is strong in chemistry but is restricted by physical diffusion.^30^ Since no better mark was found to characterize indirect chlorination, Cl release was used here.

According to the results in Section 3.1, 85% O_2_ + 15% H_2_O was the most favourable atmosphere among the three for indirect chlorination, which started at 700–800 °C. The effect of O_2_ atmosphere was similar to that of 85% O_2_ + 15% H_2_O atmosphere, but it was weaker. The results indicated that H_2_O enhanced indirect chlorination and the reaction rate of NaCl + Si/Al matrix + H_2_O was higher than that of NaCl + Si/Al matrix + O_2_; this was supported by the results of thermodynamic researches and experimental researches.^15,31,32^ H_2_O and O_2_ are usually considered as the necessary reactants for Cl release from NaCl.^23,33^ The Cl release in N_2_ atmosphere has not been paid attention to in most previous researches; however, it was proven in this research. The results indicated that indirect chlorination could occur above 1000 °C without the participation of O_2_ and H_2_O. As we know, drying process, pyrolysis/gasification process and combustion are separated on grate; thus, the atmosphere around MSW is complex and variable during incineration. The O_2_ and H_2_O-free atmosphere is available in the mechanical grate furnace. Furthermore, this finding provided important reference for decoupling indirect chlorination from direct chlorination. Overall, the positive effect of atmospheres on indirect chlorination followed the sequence of H_2_O > O_2_ > N_2_. Wang *et al.* found no Cl release in NaCl + SiO_2_ system and NaCl + Al_2_O_3_ system in air atmosphere at 850 °C; this may be due to the chlorine detecting method and the weak release amount at this temperature.^23^

### 4.2 Phase of NaCl to participate in indirect chlorination

**Fig. 6:**
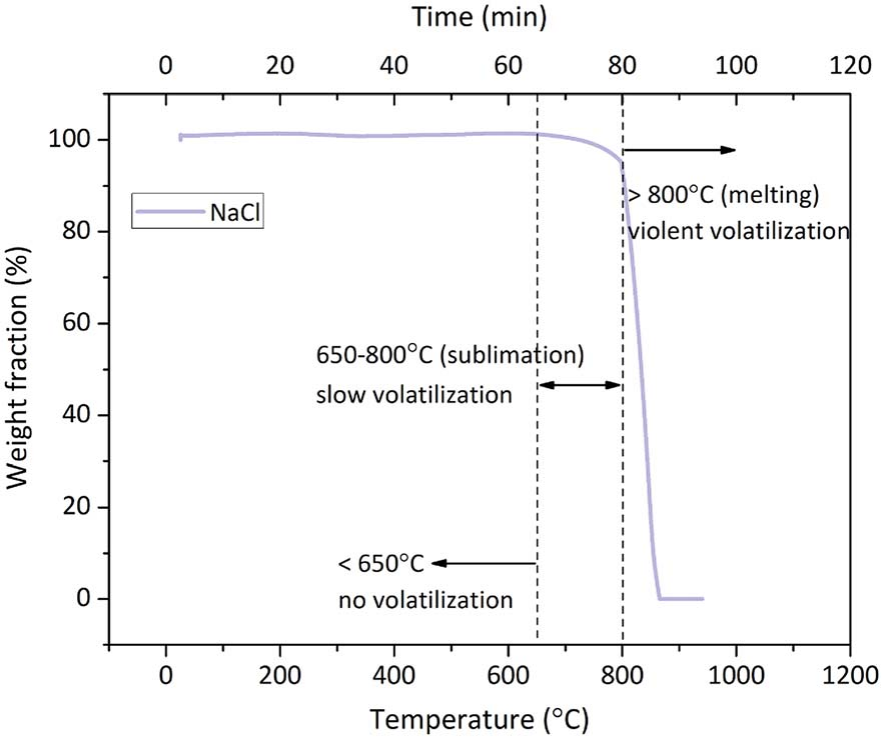
TG curve of NaCl in O_2_ atmosphere.

An interesting phenomenon is the bimodal form or the tendency at least of Cl release rate in the O_2_ and O_2_ + H_2_O atmospheres (Fig. 3). The Cl release was initiated above 700 °C, and the Cl release ratio reached the peak value at 800–900 °C; then, it decreased at 900–1000 °C and finally, it increased again at 1000–1100 °C. Such a dramatic fluctuation was considered due to the volatilization of NaCl; thus, TG test of NaCl was carried out at a heating rate of 10 °C min^−1^ and N_2_ flow rate of 100 mL min^−1^. The TG curve indicated that the sublimation of NaCl was initiated at around 650 °C, and the intense volatilization started above 800 °C, which was close to its melting point of 801 °C (Fig. 6). The sublimation of NaCl detected by TG has been reported by Matsuda *et al.*; however, it did not occur in the research of Wang *et al.*^23,33^ The bimodal tendency of Cl release rate and the NaCl volatilization fraction were compared with respect to temperature. When NaCl volatilized slightly at 650–800 °C by sublimation, the Cl release was small as well. When NaCl melted and volatilized intensely above 800 °C, the Cl release rate increased at first, then decreased, and finally increased again. According to this, vapor was considered as the main state during the indirect chlorination reaction. The Cl release was initiated at the start of NaCl sublimation; then, NaCl melted and more NaCl vapor was produced above 800 °C, resulting in the release of more Cl. This was followed by extensive melting of NaCl which caused the isolation of Si/Al matrix surfaces from O_2_ or H_2_O, inhibiting the reaction and finally, melted NaCl vaporized, resulting in intense Cl release.

### 4.3 Reactions during indirect chlorination

**Fig. 7:**
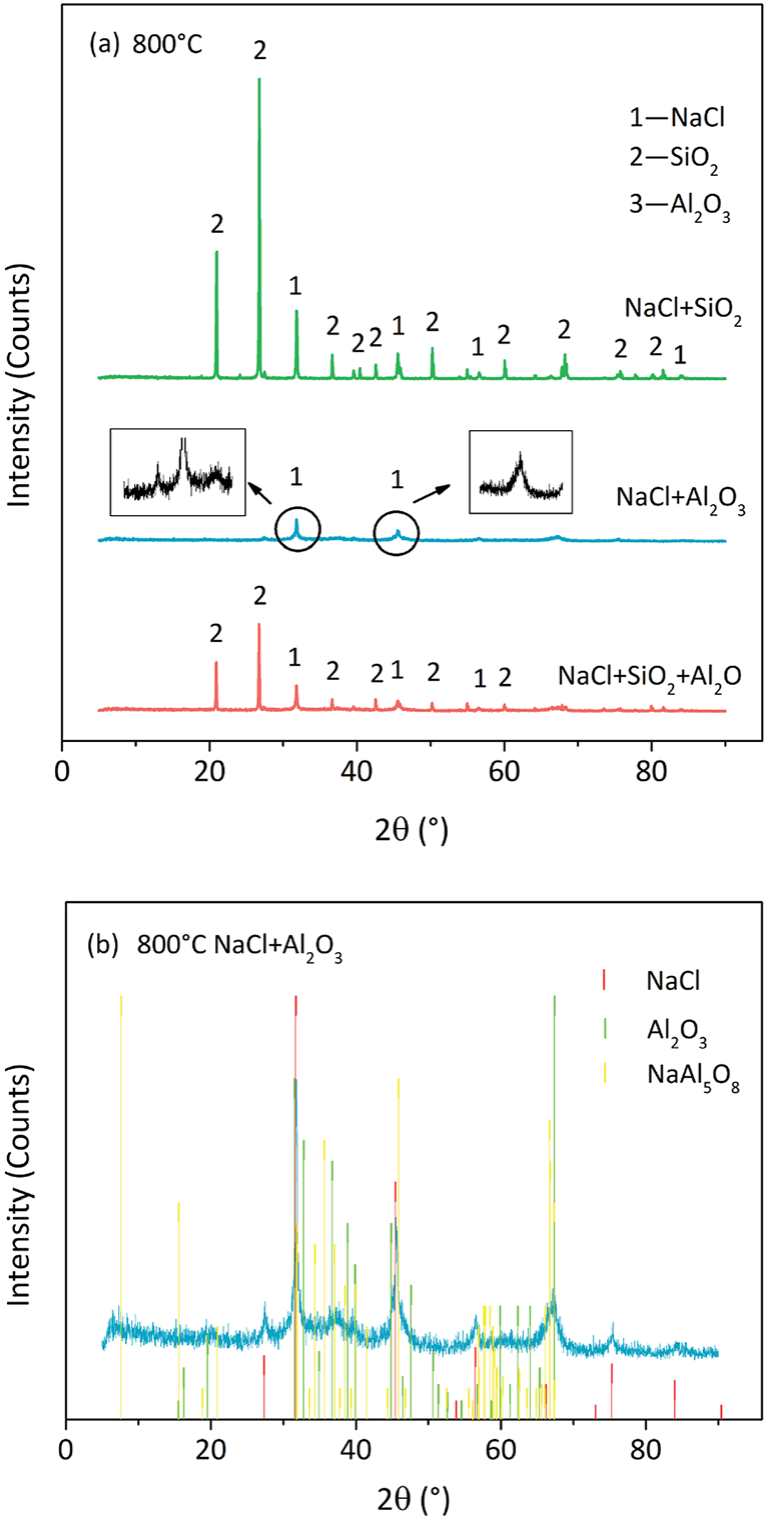
XRD patterns of residues derived from NaCl and Si/Al mixture calcination at 800 °C in 85% O_2_ + 15% H_2_O atmosphere. (a) Comparison of XRD patterns of residues derived from Na/Si/Al = 1 : 2 : 0, 1 : 0 : 2, and 1 : 1 : 1. (b) XRD pattern of residues derived from Na/Si/Al = 1 : 0 : 2.

We observed that 800 °C was the initial temperature of indirect chlorination by Cl release (Fig. 2). The initial products were analysed by the XRD detection of residues derived from sample calcination at this temperature. The atmosphere of 85% O_2_ + 25 H_2_O was the best among the three for indirect chlorination, and the corresponding XRD patterns are shown in Fig. 7(a). The residue derived from NaCl + SiO_2_ calcination was still composed of NaCl and SiO_2_ without other products. This indicated that the reaction started above 800 °C, which was similar to the results from Cl release experiments in which the Cl release was close to zero for NaCl + SiO_2_ at 800 °C. The diffraction peak intensity of NaCl in NaCl + Al_2_O_3_ case was much lower than that from NaCl + SiO_2_ calcination. This indicated that NaCl was involved in the reaction due to which less NaCl was remained. In the Cl release experiments, NaCl started to release Cl at 800 °C, which was in accordance with the results of XRD analysis. The diffraction peak intensity in the case of NaCl + SiO_2_ + Al_2_O_3_ was between that in the case of NaCl + SiO_2_ and that in the case of NaCl + Al_2_O_3_.

The XRD pattern of the residue in NaCl + Al_2_O_3_ case was indistinct in the same ordinate when compared with those of other cases. Therefore, it was plotted separately in Fig. 7(b). Al_2_O_3_ was crystallized partially at 800 °C accompanied with the formation of a small quantity of sodium aluminates. NaAl_5_O_8_ was one of the sodium aluminates, and its peak matched the diffraction peaks well. There were still some amorphous substances that were unknown. The XRD pattern of the residue in NaCl + SiO_2_ + Al_2_O_3_ case (not shown here) indicated the formation of sodium silicoaluminates. Moreover, calcinations at higher temperatures in N_2_/O_2_ atmospheres were carried out. The formations of sodium silicates, sodium aluminates and sodium silicoaluminates were found in the case of NaCl + SiO_2_, NaCl + Al_2_O_3_and NaCl + SiO_2_ + Al_2_O_3_, respectively. The formation temperatures were in accordance with Cl release temperatures. The specific compositions could not be distinguished due to the diversification of products and the disorder of XRD patterns resulting from amorphous substances.

The indirect chlorination path was surmised by the proposed reaction equations (PbO for example), where *T* indicates the initial temperature of the reaction:

(1) Cl releaseNaCl(g) + SiO_2_ + O_2_(g) → Na_*x*_Si_*y*_O_*z*_ + Cl_2_(g) *T* > 800 °CNaCl(g) + Al_2_O_3_ + O_2_(g) → Na_*x*_Al_*y*_O_*z*_ + Cl_2_(g) *T* > 800 °CNaCl(g) + SiO_2_ + Al_2_O_3_ + O_2_(g) → Na_*w*_Si_*x*_Al_*y*_O_*z*_ + Cl_2_(g) *T* > 800 °CNaCl(g) + SiO_2_ + H_2_O(g) → Na_*x*_Si_*y*_O_*z*_ + HCl(g) *T* > 800 °CNaCl(g) + Al_2_O_3_ + H_2_O(g) → Na_*x*_Al_*y*_O_*z*_ + HCl(g) *T* > 800 °CNaCl(g) + SiO_2_ + Al_2_O_3_ + H_2_O(g) → Na_*w*_Si_*x*_Al_*y*_O_*z*_ + HCl(g) *T* > 800 °CNaCl(g) + SiO_2_→Na_*x*_Si_*y*_O_*z*_ + Cl_2_(g) *T* > 1000 °CNaCl(g) + Al_2_O_3_ → Na_*x*_Al_*y*_O_*z*_ + Cl_2_(g) *T* > 1000 °CNaCl(g) + SiO_2_ + Al_2_O_3_ → Na_*w*_Si_*x*_Al_*y*_O_*z*_ + Cl_2_(g) *T* > 1000 °C

(2) ChlorinationCl_2_(g) + PbO → PbCl_2_ + O_2_(g) *T* > 800 °C or >1000 °CHCl(g) + PbO → PbCl_2_ + H_2_O(g) *T* > 800 °C

(3) VolatilizationPbCl_2_ → PbCl_2_(g) *T* > 800 °C or >1000 °C

### 4.4 Using metal volatilization to characterize direct chlorination

**Fig. 8:**
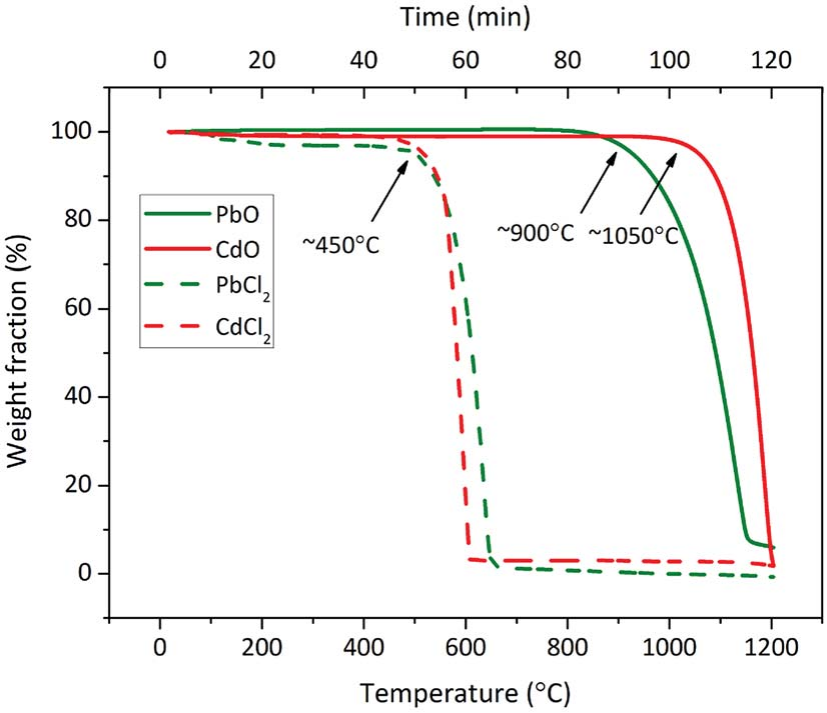
TG curves of PbO, CdO, PbCl_2_ and CdCl_2_ in N_2_ atmosphere.

The investigation of the direct chlorination of PbO and CdO was established using the characteristics of metal volatilization induced by the interaction with NaCl and Si/Al matrix. Only N_2_ atmosphere was involved for the decoupling with indirect chlorination. The accumulative volatilization fractions of Pb and Cd without NaCl addition were less than 0.3% when the temperature increased up to 1000 °C, and they were less than 4% and 1%, respectively, when the temperature increased up to 1100 °C. The volatilization above 1000 °C played a major role. The volatilization of pure substances in Fig. 8 agreed with this result. Meanwhile, the Cl release was initiated above 1000 °C in N_2_ atmosphere, which indicated that no indirect chlorination occurred below 1000 °C. Therefore, the volatilization of Pb and Cd below 1000 °C with NaCl addition in N_2_ atmosphere was irrelevant to the volatilization of PbO and CdO and indirect chlorination, but it was due to direct chlorination. In this view, the rationality of using metal volatilization to characterize direct chlorination has been proven; however, the upper limit of the temperature in this research method was only 1000 °C.

Wang *et al.* found that NaCl could chlorinate PbO without Si/Al matrix participation above 800 °C by liquid–solid reaction.^23^ Herein, the Si/Al matrix was involved in each case. Trace amounts of NaCl and PbO/CdO caused low probability of their liquid–solid contact. The volatilization of Pb and Cd below 800 °C and the effect of Si/Al matrix also contradicted this possibility. Therefore, direct chlorination was considered to occur with the participation of the Si/Al matrix.

According to the characteristics of Pb and Cd volatilization below 1000 °C in N_2_ atmosphere, the characteristics of direct chlorination were obtained. The Si/Al matrix component was found to be an important factor. When the Si/Al matrix consisted of SiO_2_ only, the initial temperature of direct chlorination was 650–700 °C. When Al_2_O_3_ was involved, it increased to 800–850 °C for Pb and 750–800 °C for Cd. The direct chlorination of Pb was faster than that of Cd. Wang *et al.* reported similar results in which the initial temperature of direct chlorination in the NaCl + PbO + SiO_2_ system was 600–611 °C and that in the NaCl + PbO + Al_2_O_3_ system was 745 °C.^23^ The lower temperature may be due to the high proportion of NaCl and PbO in the reactant system (NaCl : PbO : PbO molar ratio = 50 : 25 : 25), causing sufficient contact with reactant.^23^

### 4.5 Direct chlorination process with different Si/Al matrixes

Herein, direct chlorination was defined as the direct donation of Cl from NaCl to chlorination without Cl-containing gas release first. However, was Cl in NaCl donated to PbO/CdO directly? It was suspected that PbO/CdO reacted with the Si/Al matrix first and then, NaCl reacted with their reaction products. To determine whether this suspicion was correct, the reactions between PbO/CdO and Si/Al matrices were investigated by XRD analysis of reaction residues. PbO, CdO, SiO_2_ and Al_2_O_3_ were mixed in three ratios of 1 : 1 : 2 : 0, 1 : 1 : 0 : 2 and 1 : 1 : 1 : 1.

**Fig. 9:**
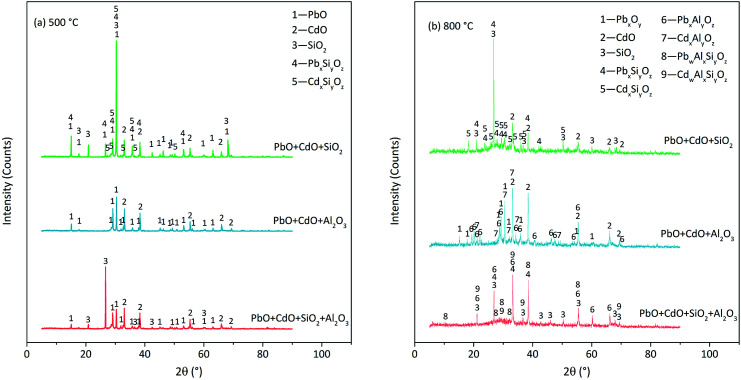
XRD patterns of residues derived from PbO, CdO and Si/Al mixture calcination at (a) 500 °C and (b) 800 °C in N_2_ atmosphere.

The XRD patterns of the residues derived at 500 °C are shown in Fig. 9. PbO/CdO reacted with SiO_2_ but not Al_2_O_3_ at 500 °C. The reaction products were lead silicates (PbSiO_3_, Pb_3_SiO_5_, *etc.*), cadmium silicates (CdSiO_3_, *etc.*) and some other substances difficult to distinguish without amorphous substance formation (Fig. 9(a)). In the PbO + CdO + Al_2_O_3_ case, at 500 °C, no lead aluminates and cadmium aluminates were found until the temperature increased up to 800 °C (Fig. 9(a) and (b)). The reaction products were lead aluminates (PbAl_2_O_4_, Pb_2_Al_2_O_5_, *etc.*), cadmium aluminates (CdAl_2_O_4_, CdAl_4_O_7_, *etc.*) and some amorphous substances. The reaction fractions of PbO and CdO in the PbO + CdO + Al_2_O_3_ case were lower than that in the PbO + CdO + SiO_2_ case because the remaining PbO and CdO were found in PbO + CdO + Al_2_O_3_ but not in the PbO + CdO + SiO_2_ case.

Consequently, the following suspicion was proven to be true: PbO/CdO reacted with the Si/Al matrix before the occurrence of direct chlorination. Moreover, the reaction between PbO/CdO and Si/Al matrices seemed to be the necessary condition for direct chlorination. In this view, it could be called pre-reaction. In the PbO + CdO + SiO_2_ case, the initial temperature of the pre-reaction was below 500 °C and the initial temperature of direct chlorination was 650–700 °C. In the PbO + CdO + Al_2_O_3_ case, the initial temperature of pre-reaction was 700–800 °C and the initial temperature of direct chlorination was 800–850 °C. This indicated that this so-called pre-reaction occurred before direct chlorination. Another evidence was that the pre-reaction fraction of CdO was lower than that of PbO, and the direct chlorination fraction of CdO was lower than that of PbO as well. Based on the analysis above, the reactions between PbO/CdO and Si/Al matrices were considered as the necessary condition of direct chlorination. In the PbO + CdO + SiO_2_+Al_2_O_3_ case, Al_2_O_3_ seemed to inhibit the reaction between PbO/CdO and SiO_2_; the pre-reaction started at 700–800 °C with the products of silicates, aluminates and aluminosilicates. The inhibition effect was due to the occupation of SiO_2_ by Al_2_O_3_ to form silicoaluminate. The reactions in O_2_ or O_2_+H_2_O atmosphere were similar to that in N_2_ atmosphere.

The direct chlorination path was surmised by the proposed reaction equations (PbO for example), where *T* indicates the initial temperature of the reaction:

(1) Pre-reactionPbO + SiO_2_ → Pb_*x*_Si_*y*_O_*z*_*T* < 500 °CPbO + SiO_2_ + O_2_ → Pb_*x*_Si_*y*_O_*z*_*T* < 500 °CPbO + Al_2_O_3_ → Pb_*x*_Al_*y*_O_*z*_*T* > 700 °CPbO + Al_2_O_3_ + O_2_ → Pb_*x*_Al_*y*_O_*z*_*T* > 700 °CPbO + SiO_2_ + Al_2_O_3_ → Pb_*w*_Si_*x*_Al_*y*_O_*z*_*T* > 700 °CPbO + SiO_2_ + Al_2_O_3_ + O_2_ → Pb_*w*_Si_*x*_Al_*y*_O_*z*_*T* > 700 °C

(2) ChlorinationNaCl + Pb_*x*_Si_*y*_O_*z*_ → PbCl_2_ + Na_*x*_Si_*y*_O_*z*_*T* > 650 °CNaCl + Pb_*x*_Al_*y*_O_*z*_ → PbCl_2_ + Na_*x*_Al_*y*_O_*z*_*T* > 800 °CNaCl + Pb_*w*_Si_*x*_Al_*y*_O → PbCl_2_ + Na_*w*_Si_*x*_Al_*y*_O_*z*_*T* > 800 °C

(3) VolatilizationPbCl_2_ → PbCl_2_(g) *T* > 650 °C or >800 °C

### 4.6 Simultaneous direct and indirect chlorination

In theory, direct chlorination and indirect chlorination should occur simultaneously in O_2_ + H_2_O atmosphere due to which the volatilization of Pb and Cd is enhanced; this was proven to be not entirely correct by the results shown in Fig. 5. In the SiO_2_-containing cases, the enhancement of chlorination indicated the occurrence of indirect chlorination. However, direct chlorination still played the main role in the whole chlorination process because the increased volatilization fraction was less than 15% for Pb and almost zero for Cd. Unexpectedly, the O_2_ + H_2_O atmosphere inhibited the direct chlorination of PbO and CdO in Al_2_O_3_-only case. With the participation of O_2_ or H_2_O, the products of PbO/CdO and Al_2_O_3_ showed lower activity to NaCl; a further investigation should be carried out to solve this. In N_2_ atmosphere, direct chlorination and indirect chlorination occur simultaneously above 1000 °C in theory. However, chlorination was weakened at this temperature (red slash area in Fig. 4), indicating that indirect chlorination did not dominate the volatilization of Pb and Cd.

## 5. Conclusions

The high-temperature chlorination of PbO and CdO induced by the interaction with NaCl and Si/Al matrix was separated into indirect chlorination and direct chlorination *via* controlling the content of reactants, reaction atmosphere and temperature.

The indirect chlorination was initiated above 800 °C in O_2_ + H_2_O atmosphere and O_2_ atmosphere and above 1000 °C in N_2_ atmosphere. The positive effect of atmospheres on indirect chlorination followed the sequence of H_2_O > O_2_ > N_2_. Compared with SiO_2_, Al_2_O_3_ had higher activity for reacting with NaCl, releasing HCl or Cl_2_. In the Cl release reaction, NaCl was in the gas phase.

The direct chlorination was initiated at 650–700 °C when the Si/Al matrix contained SiO_2_ only and at around 800 °C when the Si/Al matrix contained Al_2_O_3_ only or both SiO_2_ and Al_2_O_3_. SiO_2_ exhibited higher activity in direct chlorination than Al_2_O_3_. The pre-reaction between PbO/CdO and Si/Al matrices was considered as the necessary condition for direct chlorination. This indicated that NaCl reacted with the silicates, the aluminates and the aluminosilicates of Pb and Cd to produce PbCl_2_ and CdCl_2_.

During the chlorination in O_2_ + H_2_O atmosphere, indirect chlorination and direct chlorination occurred simultaneously, and the latter dominated Pb and Cd volatilization.

## Conflicts of interest

There are no conflicts to declare.

## Supplementary Material
